# Efficient DV-HOP Localization for Wireless Cyber-Physical Social Sensing System: A Correntropy-Based Neural Network Learning Scheme

**DOI:** 10.3390/s17010135

**Published:** 2017-01-12

**Authors:** Yang Xu, Xiong Luo, Weiping Wang, Wenbing Zhao

**Affiliations:** 1 School of Computer and Communication Engineering, University of Science and Technology Beijing (USTB), Beijing 100083, China; b20160304@xs.ustb.edu.cn; 2 Beijing Key Laboratory of Knowledge Engineering for Materials Science, Beijing 100083, China; 3 Department of Electrical Engineering and Computer Science, Cleveland State University, Cleveland, OH 44115, USA; w.zhao1@csuohio.edu

**Keywords:** wireless sensor network (WSN), received signal strength indication (RSSI), distance vector hop (DV-HOP), regularized correntropy criterion (RCC), extreme learning machine (ELM)

## Abstract

Integrating wireless sensor network (WSN) into the emerging computing paradigm, e.g., cyber-physical social sensing (CPSS), has witnessed a growing interest, and WSN can serve as a social network while receiving more attention from the social computing research field. Then, the localization of sensor nodes has become an essential requirement for many applications over WSN. Meanwhile, the localization information of unknown nodes has strongly affected the performance of WSN. The received signal strength indication (RSSI) as a typical range-based algorithm for positioning sensor nodes in WSN could achieve accurate location with hardware saving, but is sensitive to environmental noises. Moreover, the original distance vector hop (DV-HOP) as an important range-free localization algorithm is simple, inexpensive and not related to the environment factors, but performs poorly when lacking anchor nodes. Motivated by these, various improved DV-HOP schemes with RSSI have been introduced, and we present a new neural network (NN)-based node localization scheme, named RHOP-ELM-RCC, through the use of DV-HOP, RSSI and a regularized correntropy criterion (RCC)-based extreme learning machine (ELM) algorithm (ELM-RCC). Firstly, the proposed scheme employs both RSSI and DV-HOP to evaluate the distances between nodes to enhance the accuracy of distance estimation at a reasonable cost. Then, with the help of ELM featured with a fast learning speed with a good generalization performance and minimal human intervention, a single hidden layer feedforward network (SLFN) on the basis of ELM-RCC is used to implement the optimization task for obtaining the location of unknown nodes. Since the RSSI may be influenced by the environmental noises and may bring estimation error, the RCC instead of the mean square error (MSE) estimation, which is sensitive to noises, is exploited in ELM. Hence, it may make the estimation more robust against outliers. Additionally, the least square estimation (LSE) in ELM is replaced by the half-quadratic optimization technique. Simulation results show that our proposed scheme outperforms other traditional localization schemes.

## 1. Introduction

In recent years, there has been an emerging interest in the field of socially-aware computing through integrating social computing and pervasive computing [1,2]. Then, the cyber-physical social system could deeply integrate the cyber world and the physical world, as well as the social world [3]. In addition, the social sensing is a novel application of the cyber-physical social system. Additionally, a new paradigm, named cyber-physical social sensing (CPSS), was developed, in which the perception processes allow humans to participate. The wireless sensor network (WSN) with a simple architecture and cost-saving performance, plays a critical role for the sensor process and provides some important information to serve as a social network in the CPSS [4,5], where those sensors are the primary entities replacing humanity in the traditional social network [6]. Various research regarding the WSN has been undertaken [7,8,9,10,11].

Generally, a critical step in constructing a sensor network is to precisely determine the node position through the process of localization. In other words, localization is an indispensable part of WSN [12]. With the purpose of locating sensor nodes precisely, we need effective localization techniques to improve the performance of WSN. Some traditional approaches of localization, i.e., the Global Positioning System (GPS)-based method [13], manual measurement and the calibration method, are unsatisfactory sometimes due to the high cost, especially for a large-scale sensor network.

Usually, all nodes in WSN are randomly distributed, and only a few nodes, called anchor nodes, equipped with GPS, could get their positions after being scattered. However, the other nodes, called unknown nodes, do not capture their own positions. The anchors usually help those unknown nodes by using connectivity between nodes and exchanging multiple hop routing information to locate themselves. The node localization methods of sensor networks could be grouped as range-based and range-free. The former depends on angle or range measurements between nodes, which could be obtained by the time-of-arrival (TOA) method or the received signal strength indication (RSSI) method [14], and special hardware equipment is necessary. RSSI is a preferable choice due to its relatively low cost [15], and RSSI has been widely used for device-free wireless localization [16,17,18,19]. Nevertheless, RSSI is sensitive to environmental noise [19] and may lead to a decrease of localization accuracy. Meanwhile, the latter relies only on connectivity, and it is naturally less expensive and simpler [20].

Among the available range-free localization schemes, some are heuristic and simple and could be carried out in a distributed environment. Additionally, the classic range-free localization algorithm, i.e., distance vector hop (DV-HOP) [21], is a good choice in a hardware support-limited environment because of its simplicity in implementation. However, the positioning accuracy will be greatly reduced when the node distribution is uneven. Consequently, some novel methods have been proposed on the basis of DV-HOP to enhance the accuracy of localization [22,23,24].

Taking advantage of both the range-free method and the range-based method, some algorithms were proposed by incorporating RSSI and DV-HOP to execute the localization for unknown nodes [25,26,27,28]. In this way, the localization error of the unknown and anchor nodes could be reduced effectively. Nevertheless, the calculation of the coordinate may still not be accurate in some cases [29].

Motivated by the scheme of neural network (NN)-based node localization with RSSI and hop counts [30], we present a novel DV-HOP localization scheme with RSSI and regularized correntropy criterion (RCC)-based ELM (ELM-RCC), named RHOP-ELM-RCC, to improve the performance of WSN in the CPSS. Compared with SNR (signal to noise ratio) parameters, the parameters of RSSI are more related to position [31]; RSSI may be accordingly more appropriate in our proposed scheme. We combine the DV-HOP and RSSI to reduce distance measurement error without additional hardware, in which RSSI estimates the distance utilizing the decreasing degree of the signals in the transfer process [32].

Since ELM is an effective NN learning algorithm with fast learning speed and minimal human intervention [33,34], it can be used to improve the performance of WSN [35,36]. In our previous work [37], we exploited the ELM-based single-hidden layer feedforward network (SLFN) to calculate the sub-anchor nodes. Moreover, RCC could be used to improve the ability of the anti-noise of ELM [38]. Here, we utilize the algorithm ELM-RCC to calculate the coordinates of unknown nodes in this article. Then, integrating the ELM-RCC into the DV-HOP localization algorithm with RSSI, the robustness for environmental noise and transport errors may be improved.

It should be indicated that RHOP-ELM-RCC is a general schema for wireless networks, e.g., IEEE 802.11, Scenario 4G/5G. Our work assumes a well-known transmission power. The case of unknown transmission power is out of the scope of the current article. This scenario is studied in [39]. In addition, the transmission power attenuation is directly proportional to the transmission distance.

The rest of this article is as follows. Preliminaries, including DV-HOP, RSSI, ELM, correntropy and ELM-RCC, are analyzed in Section 2. The details of our proposed scheme are shown in Section 3, in which DV-HOP localizations with RSSI based on ELM or ELM-RCC are described, respectively. The simulation results and analysis are provided in Section 4. Additionally, a conclusion is drawn in Section 5.

## 2. Preliminaries

### 2.1. DV-HOP Algorithm

**Figure 1:**
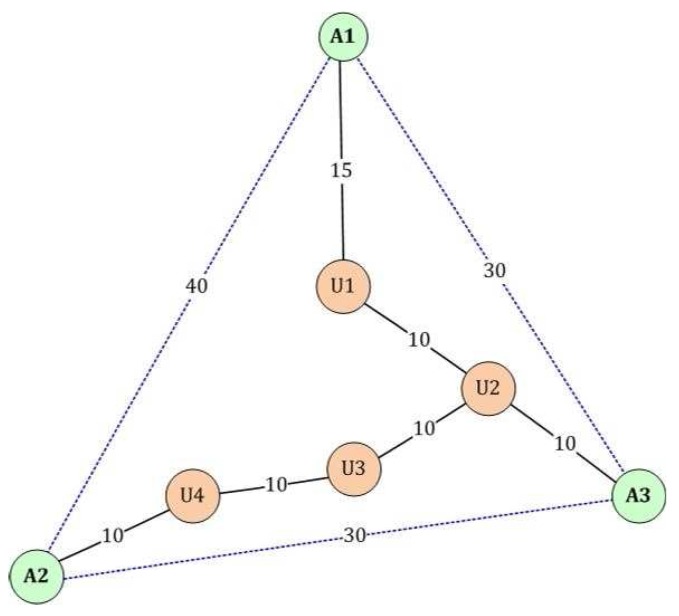
A diagram of the range measurement error for the distance vector hop (DV-HOP) algorithm.

DV-HOP is a typical range-free localization algorithm. The key of the DV-HOP scheme is that the distance between the anchor nodes and the unknown nodes is gained through multiplying the average hop size by the hop count, and then, the coordinates of the unknown nodes are obtained using the maximum likelihood estimation method [40]. The anchor node estimates the average hop size using the minimum hop count and the distances, which are gained from itself to all other anchors, and then each unknown node determines its average hop size by selecting the minimum hop count to an anchor node [41]. Although the range measurement method of DV-HOP is unaffected by environmental factors, such as landscape and climate, the range measurement error is large if the sensor nodes are unevenly distributed in WSN. For example, in Figure 1, we assume that nodes A1, A2 and A3 are all anchor nodes, and the positions of the unknown nodes U1, U2, U3 and U4 are to be identified. The true distances of these anchor nodes are known, i.e., 30, 30, 40. Then, each anchor node obtains its average hop distance as follows:

(1) $$\begin{array}{c} A1:\;\;\left(30+40\right)\times {\left(3+5\right)}^{-1}=8.75,\\ A2:\;\;\left(30+40\right)\times {\left(4+5\right)}^{-1}=7.78,\\ A3:\;\;\left(30+30\right)\times {\left(3+4\right)}^{-1}=8.57. \end{array}$$

Then, each anchor node transfers its average hop distance by broadcasting in the form of flooding through the network, and the unknown node only receives the first value as its average hop size. Our flooding method could have a negative impact on battery-powered-only anchor nodes. Nevertheless, this aspect is out of scope of the current proposal. We take the unknown node U1 shown in Figure 1 as an example; the hop count from A1 to U1 is only one hop; thus, U1 just saves the average hop distance of A1, i.e., 8.75, as its average hop size. Then, U1 estimates the distances to all other anchors as follows:

(2) $$\begin{array}{c} U1\to A1:\;\;8.75\times 1=8.75,\\ U1\to A2:\;\;8.75\times 4=35,\\ U1\to A3:\;\;8.75\times 2=17.5. \end{array}$$

In Figure 1, it is obvious that the range measurement error is large with the DV-HOP scheme, where the actual range between A1 and U1 is 15, but not 8.75. Additionally, the large range error will make the final position error even greater by using the maximum likelihood estimation method or the triangular positioning algorithm.

### 2.2. Received Signal Strength Indication

Due to the lower implementation complexity without the need for additional hardware, RSSI becomes an attractive alternative in WSN [39]. However, RSSI is easily affected by noise and obstacles and may lead to significant estimation errors; moreover, two nodes may receive different RSSI originated from each other under an uncertain environment [30]. Currently, the signal propagation model of RSSI for WSN can be divided into three types, including the two-ray ground model, free space model and log-normal shadow model [42,43]. The first two models are applicable to some special occasions, while the last one describes the fading of signal strength and is appropriate for both outdoor and indoor environments, and it is a more general model of signal propagation. Then, the received signal power of nodes for the log-normal shadow model can be defined by [44]:

(3) $$\mathrm{RSS}\left(d\right)\left(\mathrm{dBm}\right)=P_{\mathrm{tr}}-P_{\mathrm{loss}}\left(d_{0}\right)-10\tau \log_{10}\frac{d}{d_{0}}+X_{\sigma },$$

where *d* means the distance between the sending nodes and the receiving nodes, RSS(d) indicates the received signal power of nodes located at the distance of *d*, $d_{0}$ is the referenced distance, $P_{\mathrm{tr}}$ denotes the transmitting power, $P_{\mathrm{loss}}\left(d_{0}\right)$ means the path loss for $d_{0}$, *τ* indicates loss exponent in the path and its value relies on the environment of propagation and $X_{\sigma }$ is the noise in RSSI, which is described as a Gaussian random variable with zero-mean and standard deviation *σ*.

### 2.3. Extreme Learning Machine

For *P* arbitrary different training samples $\left\{{\left(x_{i},t_{i}\right)}_{i=1}^{P}\right\}$, where $t_{i}=\left(t_{i1},t_{i2},\cdots ,t_{in}\right)\in R^{n}$ and $x_{i}$ = $(x_{i1}$, $x_{i2}$, ⋯, $x_{im})\in R^{m}$. In a WSN, $t_{i}$ denotes the coordinate of node *i*, and $x_{i}$ means the distances from the node *i* to all other *m* nodes. With a random input x, the corresponding output function of ELM could be specified as:

(4) O(x)=ℏ(x)W,

(5) $$W={\left(\frac{I}{C}+HH^{T}\right)}^{-1}H^{T}T,$$

where W indicates the output weight in the connection of the hidden layer and the output in an NN, I denotes the identity matrix, *C* is the regularization parameter, $O_{P\times n}={\left(O_{1},O_{2},\cdots ,O_{P}\right)}^{T}$, $T_{P\times n}={\left(T_{1},T_{2},\cdots ,T_{P}\right)}^{T}={\left(t_{1},t_{2},\cdots ,t_{P}\right)}^{T}$, $H_{P\times h}={\left(\hbar \left(x_{1}\right),\hbar \left(x_{2}\right),\cdots ,\hbar \left(x_{P}\right)\right)}^{T}$ represents the output of the hidden layer corresponding to the given training dataset and *h* indicates the amount of hidden nodes in the hidden layer. In addition, $\hbar \left(x_{i}\right)=\left(\hbar_{1}\left(x_{i}\right),\hbar_{2}\left(x_{i}\right),\cdots ,\hbar_{h}\left(x_{i}\right)\right)\in R^{h}$, where i=1,2,⋯,P, and $\hbar (x_{i})$ maps corresponding input $x_{i}$ from *m*-dimensional space to the *h*-dimensional feature space of the hidden layer.

If the feature mapping function ℏ(x) in the hidden layer is unclear to users, a kernel-based ELM was proposed in [33]. Then, the kernel matrix of ELM could be defined as:

(6) $$\begin{array}{c} \Omega_{\mathrm{ELM}}=HH^{T}=\left[\begin{array}{c} \hbar (x_{1})\\ \vdots \\ \hbar (x_{P}) \end{array}\right]\left[\begin{array}{ccc} \hbar^{T}\left(x_{1}\right) & \cdots & \hbar^{T}\left(x_{P}\right) \end{array}\right]\\ \;\;\;\;\;\;\;\;\;\;=\left[\begin{array}{ccc} \begin{array}{c} \hbar \left(x_{1}\right)\hbar^{T}\left(x_{1}\right)\\ \vdots \\ \hbar \left(x_{P}\right)\hbar^{T}\left(x_{1}\right) \end{array} & \begin{array}{c} \cdots \\ \vdots \\ \cdots \end{array} & \begin{array}{c} \hbar \left(x_{1}\right)\hbar^{T}\left(x_{P}\right)\\ \vdots \\ \hbar \left(x_{P}\right)\hbar^{T}\left(x_{P}\right) \end{array} \end{array}\right]\\ \;\;\;\;\;\;\;\;\;\;=\left[\begin{array}{ccc} \begin{array}{c} K(x_{1},x_{1})\\ \vdots \\ K(x_{P},x_{1}) \end{array} & \begin{array}{c} \cdots \\ \vdots \\ \cdots \end{array} & \begin{array}{c} K(x_{1},x_{P})\\ \vdots \\ K(x_{P},x_{P}) \end{array} \end{array}\right], \end{array}$$

where the corresponding kernel function in Equation (6) could be denoted as:

(7) $$\begin{array}{c} K\left(a,b\right)=e^{-\gamma ||a-b|{|}^{2}}, \end{array}$$

where *γ* is parameter of the kernel.

Then, the output function in Equation (4) could be rewritten as:

(8) $$\begin{array}{c} O\left(x\right)=\hbar \left(x\right){\left(\frac{I}{C}+\Omega_{\mathrm{ELM}}\right)}^{-1}H^{T}T. \end{array}$$

In the above kernel implementation of ELM, it is not necessary to explicitly give the formula of the feature mapping function in the hidden layer, as well as the number of hidden nodes [45].

The weight connecting matrix W between the output layer and the hidden layer in Equation (4) is obtained by [38]:

(9) $$\begin{array}{c} \min_{W}\left({∥O-T∥}_{F}^{2}\right)=\min_{W}\left({∥\mathrm{HW}-T∥}_{F}^{2}\right), \end{array}$$

where ${∥\cdot ∥}_{F}$ indicates the Frobenius norm. Usually, the minimum norm least square problem is sensitive to noises.

Equality-constrained optimization is used to enhance the stability and generalization of ELM [33]. Hence, Equation (9) could be converted as:

(10) $$\begin{array}{c} \min_{W}\left({∥\mathrm{HW}-T∥}_{F}^{2}+\lambda {∥W∥}_{F}^{2}\right), \end{array}$$

where ${∥W∥}_{F}^{2}=\sum_{i=1}^{h}{∥W_{i}∥}_{2}^{2}$, in which the $L_{2}$-norm is used, and *λ* is the regularization parameter, usually, $\lambda =\frac{I}{C}$. Here, *C* and I are mentioned above.

Thus, the kernel-based ELM with regularization could be described as Algorithm 1.
**Algorithm 1** The kernel-based ELM with regularization.Input: training samples {($x_{i}$, $t_{i}$) |$x_{i}\in R^{m}$, $t_{i}\in R^{n}$, i=1,⋯,P}; the regularization parameter *C*; the kernel function K(a,b); the input of a random testing sample x.  (1) Calculate the kernel matrix $\Omega_{\mathrm{ELM}}$ of the given *P* training samples based on Equation (6);  (2) Calculate the output of the test sample O based on Equation (8). Output: the test sample O.

### 2.4. Correntropy

Inspired by information theoretic learning (ITL) [46], the original correlation function of correntropy [47] is extended to the general case [48], while it is only used for a single random process before. Assume two arbitrary random variables *S* and *J*, of which the similarity could be measured by correntropy:

(11) $$\begin{array}{c} V_{\delta }\left(S,J\right)=E\left[\kappa_{\delta }\left(S-J\right)\right], \end{array}$$

where $\kappa_{\delta }$ denotes the kernel function defined by Mercer’s theorem [49] and the mathematical expectation denoted by E[·]. Then, the maximum of (11) is named the maximum correntropy criterion (MCC) [48,50].

Let *P* be the number of samples and it is finite, then the correntropy could be expressed as:

(12) $$\begin{array}{c} {\hat{V}}_{\delta }\left(S,J\right)=\frac{1}{P}\sum_{i=1}^{P}\kappa_{\delta }\left(s_{i}-j_{i}\right), \end{array}$$

we omit the subscript *δ* in $\kappa_{\delta }$ for simplicity, and:

(13) $$\begin{array}{c} \kappa \left(s_{i}-j_{i}\right)=\kappa_{\delta }\left(s_{i}-j_{i}\right)=e^{-\frac{{\left(s_{i}-j_{i}\right)}^{2}}{2\delta^{2}}}. \end{array}$$

Since correntropy is robust against outliers, it outperforms the traditional measure, i.e., mean squared error (MSE), when there exist outliers in the training data [48].

### 2.5. Regularization Correntropy Criterion-Based ELM

Another optimal solution of Equation (9) could be obtained by utilizing MCC [38], which is defined as:

(14) $$\begin{array}{c} F\left(\tilde{W}\right)=\max_{\tilde{W}}\sum_{i=1}^{P}\kappa \left(T_{i}-O_{i}\right), \end{array}$$

where the target vector, $T_{i}$, corresponds to the *i*-th input vector $x_{i}$, while $O_{i}$ is obtained by [38]:

(15) $$\begin{array}{c} O_{i}=\sum_{j=1}^{h}W_{j}\hbar_{j}\left(x_{i}\right)+\omega_{0},\;\;i=1,2,\cdots ,P, \end{array}$$

where $W_{j}={\left(W_{j1},W_{j2},\cdots ,W_{jn}\right)}^{T}\in R^{n}$ and the bias vector $\omega_{0}={\left(\omega_{01},\omega_{02},\cdots ,\omega_{0n}\right)}^{T}\in R^{n}$ represents the threshold of the output units.

For simplicity, we give a definition $\tilde{\hbar }\left(x_{i}\right)=\left({\tilde{\hbar }}_{1}\left(x_{i}\right),{\tilde{\hbar }}_{2}\left(x_{i}\right),\cdots ,{\tilde{\hbar }}_{h+1}\left(x_{i}\right)\right)=\left(1,\hbar_{1}\left(x_{i}\right),\hbar_{2}\left(x_{i}\right),\cdots ,\hbar_{h}\left(x_{i}\right)\right)\in R^{h+1},\;i=1,2,\cdots ,P$. Additionally, ${\tilde{H}}_{P\times (h+1)}={\left(\tilde{\hbar }\left(x_{1}\right),\tilde{\hbar }\left(x_{2}\right),\cdots ,\tilde{\hbar }\left(x_{P}\right)\right)}^{T}$. In addition, ${\tilde{W}}_{(h+1)\times n}={\left({\tilde{W}}_{1},{\tilde{W}}_{2},\cdots ,{\tilde{W}}_{h+1}\right)}^{T}={\left(\omega_{0},W_{1},\cdots ,W_{h}\right)}^{T}$, where ${\tilde{W}}_{j}=\left({\tilde{W}}_{j1},{\tilde{W}}_{j2},\cdots ,{\tilde{W}}_{jn}\right)\in R^{n},\;j=1,2,\cdots ,\left(h+1\right)$. Then, Equation (15) could be updated as:

(16) $$\begin{array}{c} O_{i}=\sum_{j=1}^{h+1}{\tilde{\hbar }}_{j}\left(x_{i}\right){\tilde{W}}_{j}. \end{array}$$

Then, we add $L_{2}$ regularization into Equation (14), named RCC, and it is revised as:

(17) $$\begin{array}{c} F\left(\tilde{W}\right)=\max_{\tilde{W}}\left[\sum_{i=1}^{P}\kappa \left(T_{i}-\sum_{j=1}^{h+1}{\tilde{\hbar }}_{j}\left(x_{i}\right){\tilde{W}}_{j}\right)-\lambda {∥\tilde{W}∥}_{F}^{2}\right], \end{array}$$

where *λ* still means the regularization parameter. Here, unlike the optimization in the kernel-based ELM with regularization using least square estimation (LSE), the half-quadratic optimization method [51] and the position rules of the convex conjugated function [52,53,54] are exploited to get the optimal solution [38]. With a fixed $\tilde{W}$, Equation (17) is converted to:

(18) $$\begin{array}{c} F\left(\tilde{W}\right)=F\left(\tilde{W},\theta \right)=\max_{\tilde{W},\theta }\left[\sum_{i=1}^{P}\left(\theta_{i}\frac{{∥T_{i}-\sum_{j=1}^{h+1}{\tilde{\hbar }}_{j}\left(x_{i}\right){\tilde{W}}_{j}∥}_{2}^{2}}{2\delta^{2}}-\varphi \left(\theta_{i}\right)\right)-\lambda {∥\tilde{W}∥}_{F}^{2}\right], \end{array}$$

and in half-quadratic optimization, $\theta =(\theta_{1},\theta_{2},\cdots ,\theta_{P})$ represents the auxiliary variables.

Let D be a diagonal matrix, where the one in the *i*-th column and the *i*-th row is $D_{ii}$ = $\theta_{i}^{r+1}$. Then, we have an iterative process as follows [38]:

(19) $$\begin{array}{c} \theta_{i}^{r+1}=-\kappa \left(T_{i}-\sum_{j=1}^{h+1}{\tilde{\hbar }}_{i}\left(x_{j}\right){\tilde{W}}_{j}^{r}\right), \end{array}$$

(20) $$\begin{array}{c} {\tilde{W}}^{r+1}=\underset{\tilde{W}}{\mathrm{argmax}}\left[\mathrm{Tr}\left({\left(T-\tilde{H}\tilde{W}\right)}^{T}D\left(T-\tilde{H}\tilde{W}\right)-\lambda {\tilde{W}}^{T}\tilde{W}\right)\right], \end{array}$$

where *r* means the *r*-th iteration and it is determined by:

(21) $$\begin{array}{c} |C_{r}-C_{r+1}|<\varepsilon . \end{array}$$

Here, *ε* is a user-specified threshold, which controls the number of iterations indirectly. In addition, C is the correntropy of the target vector and the estimated output vector, and it is given as:

(22) $$\begin{array}{c} C=\frac{1}{P}\sum_{i=1}^{P}\kappa \left(T_{i}-O_{i}\right). \end{array}$$

## 3. The Proposed Scheme

This section is to demonstrate the details of the novel scheme of DV-HOP localization with RSSI based on ELM-RCC, named RHOP-ELM-RCC. RSSI is used to improve the localization accuracy, and the accuracy could be further improved if the noise sensitivity of RSSI could be overcome. Moreover, when there exist outliers, the estimated coordinates, calculated by least squares (LS) [55] or ELM [37], will be affected to a certain extent. Then, the ELM-RCC mentioned above could decrease the effect of both noise and transport error. Meanwhile, for the sake of comparison, we also give a description of the DV-HOP localization algorithm with RSSI based on ELM, named RHOP-ELM.

### 3.1. DV-HOP Localization with RSSI Based on ELM-RCC

In the proposed scheme, we reduce distance measurement error by incorporating the DV-HOP and RSSI without additional hardware. After getting the distance from all of the anchor nodes to each unknown node, the coordinates of unknown nodes are calculated by the SLFN using ELM-RCC, which is of good nonlinear mapping capability and high learning speed, as well as it shows a powerful noise-resistant ability. Here, the localization scheme RHOP-ELM-RCC is presented as follows.

(1) Each anchor node delivers a beacon detail and RSSI packet to all neighboring nodes through broadcasting. The beacon message includes the identity ${\mathrm{ID}}_{i}$ of the anchor node, location coordinates $\left(x_{i},y_{i}\right)$, hop count value ${\mathrm{hops}}_{i}$ initialized to zero and the accumulated distance ${\mathrm{DRSSI}}_{i}$, which is initialized to zero, as well. Then, the format of the beacon message can be expressed as $\left\{{\mathrm{ID}}_{i},\left(x_{i},y_{i}\right),{\mathrm{hops}}_{i},{\mathrm{DRSSI}}_{i}\right\}$. When each neighboring node receives the broadcast, it updates the values of ${\mathrm{hops}}_{i}$ and ${\mathrm{DRSSI}}_{i}$ through Equation (23) and then continues to broadcast the updated beacon message to other neighbor nodes.
  (23) $$\left\{\begin{array}{c} {\mathrm{hops}}_{i}={\mathrm{hops}}_{i}+1\\ {\mathrm{DRSSI}}_{i}={\mathrm{DRSSI}}_{i}+\mathrm{dis}\_\mathrm{hop} \end{array}\right.$$
  where “dis_hop” is the estimated distance transformed by the RSSI value of this neighboring node [56]. Note that the increase of the number of RSSI samples could reduce the impact of noise on RSSI measurement. Thus, given a certain number of RSSI samples, we average all of the RSSI values in the same measurement. Then, “dis_hop” can be defined by:
  (24) $$\mathrm{dis}\_\mathrm{hop}=10^{\frac{P_{\mathrm{tr}}-P_{\mathrm{loss}}\left(d_{0}\right)-\bar{\mathrm{RSSI}}}{10\tau }}\times d_{0},$$
  where $\bar{\mathrm{RSSI}}$ is the average RSSI values of this neighboring node.
  A node will compare the newly arriving ${\mathrm{hops}}_{i}$ with the existing ${\mathrm{hops}}_{i}$ once it receives a new packet of the same ID and will discard the new message of which the hop count is greater than the existing hop count. Otherwise, the new message would be adopted to replace the existing message of the same ID. After this process, all nodes in the framework will get the minimal hop count and the corresponding accumulated RSSI distance to every anchor node.
(2) Once the minimal hop count of one anchor and the corresponding accumulated RSSI distances from the anchor to other anchors are obtained, naturally, an average hop size and RSSI range of one hop could be estimated easily. The average hop size written as ${\mathrm{HOP}}_{i}$ and average RSSI distance written as ${\mathrm{DRSSAVG}}_{i}$ per hop are then estimated by anchor node *i* as:
  (25) $$\left\{\begin{array}{c} {\mathrm{HOP}}_{i}=\left(\sum_{j\neq i}^{n}\sqrt{{\left(x_{i}-x_{j}\right)}^{2}+{\left(y_{i}-y_{j}\right)}^{2}}\right)\cdot {\left(\sum_{j\neq i}^{n}h_{ij}\right)}^{-1}\\ {\mathrm{DRSSAVG}}_{i}=\left(\sum_{j\neq i}^{n}{\mathrm{DRSSI}}_{ij}\right)\cdot {\left(\sum_{j\neq i}^{n}h_{ij}\right)}^{-1} \end{array}\right.$$
  where $\left(x_{j},y_{j}\right)$ and $\left(x_{i},y_{i}\right)$ are the coordinates of anchor *j* and anchor *i*, respectively. Additionally, $h_{ij}$ is the minimum hop count between anchor node *i* and anchor node *j*; ${\mathrm{DRSSI}}_{ij}$ is the RSSI accumulated distance between anchor *i* and anchor *j*. Here, the number of anchor nodes is *n*.
  After obtaining the average hop size and the average hop RSSI distance, each anchor node transfers its hop size and average hop RSSI distance information. Once the unknown node gets the average hop RSSI range information from a certain anchor, as well as the hop size, it saves them as the average hop RSSI distance and the average hop size, then omits all of the subsequent information. Obviously, such a strategy guarantees that most of the unknown nodes will only receive the average hop size and average RSSI distance for one hop of the closest anchor nodes with the minimal hops.
(3) The correction factor *γ* is estimated for the size of each hop through dividing the RSSI distance per hop, dis_hop, by average RSSI distance per hop. Then, the correction hop size can be updated by multiplying the correction factor by the average hop size. Let *m* be the hop count of unknown node *j* and anchor *i*. Then, the distance between the anchor *i* and unknown node *j* could be gained by:
  (26) $$\begin{array}{c} d_{ji}=\sum_{k=1}^{m}\gamma_{k}\times {\mathrm{HOP}}_{i}=\sum_{k=1}^{m}\frac{{\mathrm{dis}\_\mathrm{hop}}_{k}}{{\mathrm{DRSSAVG}}_{i}}\times {\mathrm{HOP}}_{i}\\ \;\;\;\;\;\;=\frac{\sum_{k=1}^{m}{\mathrm{dis}\_\mathrm{hop}}_{k}}{{\mathrm{DRSSAVG}}_{i}}\times {\mathrm{HOP}}_{i}=\frac{{\mathrm{DRSSI}}_{ji}}{{\mathrm{DRSSAVG}}_{i}}\times {\mathrm{HOP}}_{i}, \end{array}$$
  where $\gamma_{k}\;\left(k=1,\cdots ,m\right)$ means the correction factor of the *k*-th hop from anchor *i* to unknown node *j*, ${\mathrm{dis}\_\mathrm{hop}}_{k}$ means the RSSI range of the *k*-th hop and ${\mathrm{DRSSI}}_{ji}$ is the RSSI accumulated distance of anchor node *i* and unknown node *j*. From Equation (26), each unknown node could evaluate the distances to all anchor nodes on the basis of the stored information in packets, which include the accumulated RSSI distance to each anchor node, its own hop size and average RSSI distance per hop.
(4) When the estimated distances from each anchor node to the unknown nodes are obtained, we will use the SLFN based on ELM-RCC to obtain the coordinates of these unknown nodes. The training samples for the SLFN using ELM-RCC are obtained from the virtual framework covering all cases [30]. If all nodes are deployed randomly in an N×N area, N×N training samples could be easily obtained. The inputs of these training datasets are the distances between every two coordinates from (1,1),(1,2)⋯,(1,N),⋯,(N,1), (N,2),⋯,(N,N), in the virtual complete topology to all anchor nodes, and the outputs of these samples are their corresponding coordinates. After getting the training samples, the SLFN using ELM-RCC is accordingly constructed and trained by using these N×N training samples and learning algorithm ELM-RCC, then the coordinate of the unknown node *j* could be estimated by exploiting the trained SLFN on the basis of input vector $d_{j}={\left(d_{jl}\right)}_{l=1}^{q}$, where *q* is the number of anchors, $d_{jl}$ is the distance between unknown node *j* and anchor *l*, which could be obtained using Equation (26).

On the whole, the proposed DV-HOP localization scheme using RSSI and ELM-RCC could be concluded in the Algorithm 2.
**Algorithm 2** DV-HOP localization scheme with RSSI based on ELM-RCC (RHOP-ELM-RCC).Input: the distance between anchor *l* and unknown node *j*: $d_{jl}\;\left(l=1,2,\cdots ,q\right)$.  (1) Obtain the hop count and RSSI distance by broadcasting the beacon messages and RSSI packets of each anchor nodes;  (2) Average hop size and calculate the mean value of RSSI distance per hop based on Equation (25);  (3) Compute the distances between anchor nodes and unknown nodes with the correction factor *γ* on the basis of Equation (26);  (4) Use the distances obtained in Equation (26) as the input of ELM-RCC, and then, calculate the coordinate $\left(x_{j},y_{j}\right)$ for unknown node *j* using ELM-RCC. Output: the coordinate of unknown node *j*.

### 3.2. DV-HOP Localization Scheme with RSSI Using Kernel-Based ELM

The DV-HOP localization scheme with RSSI using kernel-based ELM (RHOP-ELM) is also presented here to compare with RHOP-ELM-RCC. The only difference between them is whether RCC is exploited. The general steps of RHOP-ELM are similar to RHOP-ELM-RCC, and we could get RHOP-ELM by using the kernel-based ELM with regularization to replace the ELM-RCC in Step (4) of Algorithm 2.

## 4. The Performance Comparison and Analysis

### 4.1. Simulation Description

To test the performance of RHOP-ELM-RCC and RHOP-ELM in a WSN, some simulations are carried out. We measure the effectiveness of those schemes through localization error. Meanwhile, our performance comparisons are implemented among those schemes, including the DV-HOP scheme [24], DV-HOP utilizing RSSI (named RHOP) [29], a new DV-HOP (named One-HOP) [41], RHOP-ELM and RHOP-ELM-RCC. The MATLAB R2012a computing environment is applied to all simulations.

The training samples are obtained from the virtual framework including all cases. In this article, WSN is deployed in a two-dimensional area, and the actual data samples are randomly gathered from 50 nodes of the area of 50m×50m or 100 nodes from an area of 100m×100m. Then, we proportionally select the anchor nodes from all nodes. It should be indicated that the node density of the area of 100m×100m with 100 nodes is 0.01, while the node density is 0.02 in the area of 50m×50m with 50 nodes. Additionally, it explains why in our simulations, the localization error in the area 100m×100m with 100 nodes is higher than that in the area 50m×50m.

**Figure 2:**
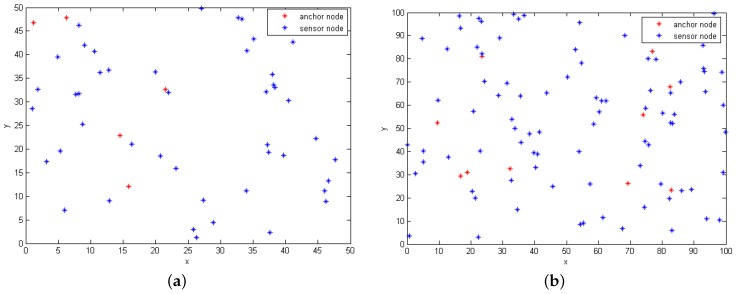
Different areas of node distribution. (**a**) There are 50 nodes in the area of 50 m × 50 m; (**b**) There are 100 nodes in the area of 100 m × 100 m.

**Figure 3:**
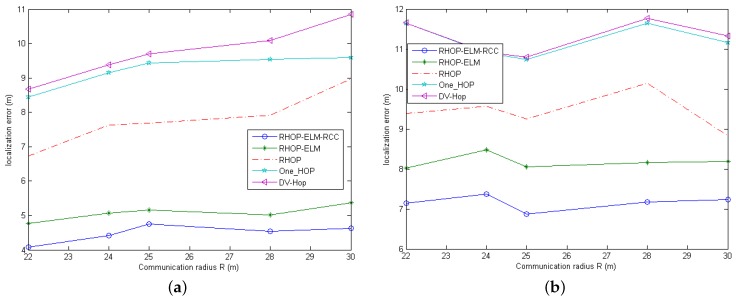
The impact of *R* in different areas. (**a**) The location error against *R* in the area of 50 m × 50 m; (**b**) The location error against *R* in the area of 100 m × 100 m.

Moreover, the sensor nodes and anchor nodes could communicate freely, and they have the same communication capabilities. In the following simulations, we assume that 10% of the total nodes are anchor nodes, as shown in Figure 2. Additionally, every two nodes could communicate on the condition that the Euclidean distance between those two nodes is within the node transmission range *R*, which is the same as its maximum communication distance. Then, we run each algorithm 20 times in different areas and then calculate the average location error against *R*, (R∈{22,24,25,28,30}) shown in Figure 3; finally, we set R=25. In the simulations of DV-HOP, RHOP and one-HOP, if there exist less than three anchor nodes that could communicate with an unknown node within the communication range *R*, then the coordinate of the unknown node cannot be obtained.

**Figure 4:**
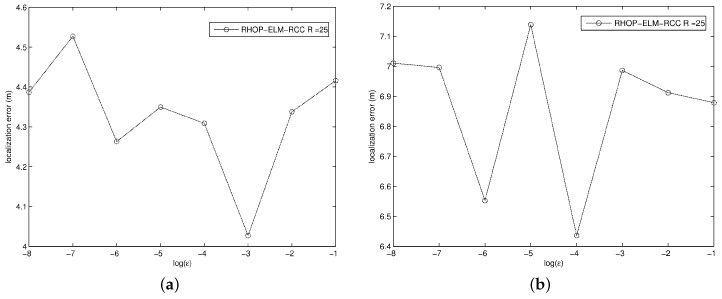
The impact of *ε* in different areas when R=25. (**a**) The average *ε* in the area of 50 m × 50 m when R=25; (**b**) The average *ε* in the area of 100 m × 100 m when R=25.

Additionally, other simulation parameters for the algorithms in the network are shown in Table 1. The parameter *ε* of the proposed algorithm affects the location accuracy to a certain extent. We execute the proposed algorithm 20 times within different area; *ε* is set in $\left\{10^{-1},10^{-2},10^{-3},10^{-4},10^{-5},10^{-6},10^{-7},10^{-8}\right\}$, and the average value is shown in Figure 4. To obtain low location error, we accordingly set $\varepsilon =10^{-3}$ in the area of 50m×50m and $\varepsilon =10^{-4}$ in the area of 100m×100m.

Here, the performance measurement is the localization error, which is inversely proportional with the localization accuracy. Then, the localization error is mathematically modeled as:

(27) $$\begin{array}{c} \mathrm{Error}={\left(\frac{1}{m}\sum_{i=1}^{m}\left({\left(x_{i}-x_{i}^{\prime }\right)}^{2}+{\left(y_{i}-y_{i}^{\prime }\right)}^{2}\right)\right)}^{\frac{1}{2}}, \end{array}$$

where $\left(x_{i}^{\prime },y_{i}^{\prime }\right)$ and $\left(x_{i},y_{i}\right)$ are the estimated coordinate and real coordinate of unknown node *i*, respectively. Additionally, *m* means the number of the unknown nodes except those nodes whose coordinates cannot be obtained.

### 4.2. Localization Errors against the Amount of Anchor Nodes

The system scenarios with pairs of the amount of nodes and node distribution area are (50, 50m×50m) and (100, 100m×100m), with the percentage of anchor nodes varying within {10%, 20%, 30%, 40%, 50%}. In addition, the RSSI samples and the noise standard deviation are set to 10 and 5, respectively.

**Figure 5:**
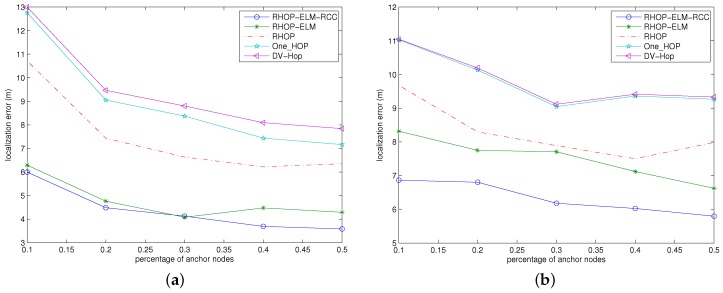
Localization errors against the amount of anchor nodes. (**a**) The case with 50 nodes in the area of 50 m × 50 m; (**b**) The case with 100 nodes in the area of 100 m × 100 m.

Figure 5 demonstrates the comparison results of our scheme with other schemes under such a condition that the number of anchor nodes changes while the amount of total nodes remains unchanged. It is clear that the accuracy rate of the localization algorithm is closely correlated with the density of anchor nodes, and our scheme outperforms other schemes in localization error. Specifically, RHOP-ELM-RCC is superior to RHOP-ELM. When the localization errors of all schemes are large, the ratio of anchor nodes is very small; while increasing the ratio of anchor nodes, the localization error of each scheme decreases. The reason is that the growth of the percentage of anchor nodes will reduce the distance between unknown nodes and the anchor nodes, decrease the information loss and lead to a relatively high accuracy.

### 4.3. Localization Errors against the Amount of RSSI Samples

Here, the WSN scenario is with a total of 50 nodes in 50m×50m and 100 nodes in 100m×100m. In addition, the anchor density is fixed to 20%, and the noise standard deviation is supposed to be five. Besides, the number of RSSI samples is described in Table 1.

**Figure 6:**
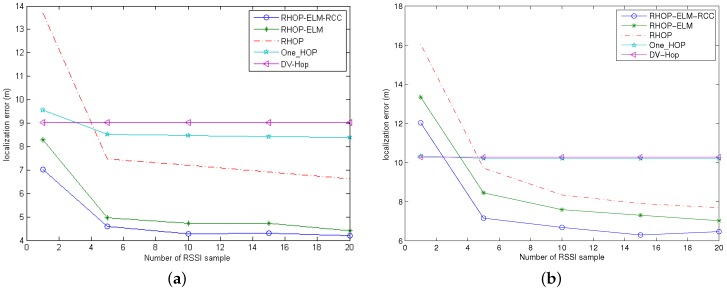
Localization errors against the amount of RSSI samples. (**a**) The case with 50 nodes in the area of 50 m × 50 m; (**b**) The case with 100 nodes in the area of 100 m × 100 m.

We observe that increasing RSSI samples is able to achieve an improvement in the distance estimation. Meanwhile, when the number of RSSI samples increases to 10 in Figure 6a and 15 in Figure 6b, the tendencies of localization error approach a steady state. These indicate that the number of RSSI samples is not a critical influencing factor in the positioning error along with the increase of this parameter. Additionally, the curves of DV-HOP and one-HOP are nearly horizontal, and the reason is that RSSI is not adopted in DV-HOP, which only utilizes hop count, while one-HOP does not exploit RSSI unless the hop count between the anchor node and the unknown node is just one. Besides, our proposed scheme is much better than others, and the performance of RHOP-ELM-RCC is also superior to that of RHOP-ELM.

### 4.4. Localization Errors against the Noise Standard Deviation

During this simulation, the density of anchors and the amount of RSSI sample are set to 20% and 10, respectively. Additionally, the dynamic standard deviation of noise could be found in Table 1. The simulation performs on two different topology areas with different total nodes, as well as different communication ranges, which are described in Section 4.1.

**Figure 7:**
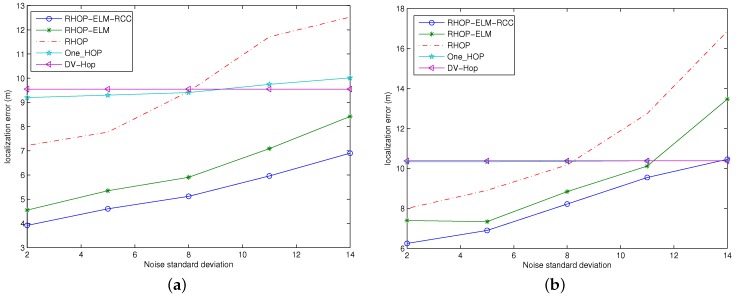
Localization errors against the noise standard deviation. (**a**) The case with 50 nodes in the area of 50 m × 50 m; (**b**) The case with 100 nodes in the area of 100 m × 100 m.

The localization errors against different noise standard deviations are shown in Figure 7. Obviously, DV-HOP and one-HOP are less effected by the noise. Because DV-HOP does not depend on the RSSI and RSSI is only being used when hop count between the unknown node and an anchor node is right at one in the one-HOP scheme, when the hop count is over one, one-HOP works similarly to DV-HOP, which is unrelated to the RSSI. In addition, the other schemes are implemented by using RSSI through the whole process.

It should be indicated that the increase of noise standard deviation would lead to unstable fluctuating of RSSI in the log-normal shadow model during the calculation of the accuracy of the distance estimation. The localization errors of schemes related to RSSI all increase along with the increase of noise deviation. Comparing the performance of RHOP, RHOP-ELM and RHOP-ELM-RCC, the contribution of ELM could help to reduce the localization error to a certain extent, and combining RCC with half-quadratic optimization could further improve anti-noise ability.

### 4.5. Localization Errors against the Outliers

In this part, we will demonstrate the robustness against outliers, caused by transmission error. In this simulation, the density of anchors is 20%; the number of RSSI samples is 10; the standard deviation of noise is five; and other initial parameters are shown in Table 1.

Since different algorithms have different ways to calculate the coordinates of unknown nodes, as well as the methods to generate outliers, to generate abnormal values for RHOP-ELM-RCC and RHOP-ELM, we randomly choose a proportion of outputs in the training samples, i.e., the coordinates of unknown nodes in the training samples, and change their coordinates. Meanwhile, for other schemes, i.e., DV-HOP, one-HOP and RHOP, we randomly select a proportion of sensor nodes as outliers, after the corresponding RSSI distances and hop counts are obtained, then change their coordinates. Additionally, the proportion of outliers is chosen from {0,3%,6%,9%,12%,15%,18%}.

**Figure 8:**
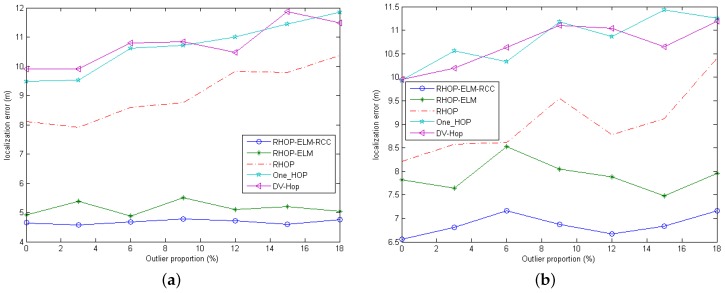
Localization errors against the outliers. (**a**) The case with 50 nodes in the area of 50 m × 50 m; (**b**) The case with 100 nodes in the area of 100 m × 100 m.

Figure 8 illustrates that, except the location error of RHOP-ELM-RCC, the location errors of other schemes are influenced by the outliers, and the trends of location error increase along with the increase of the proportion of outlier. Although there exist outliers, our proposed scheme outperforms others, due to the utilization of robust correntropy.

## 5. Conclusions

In the wireless CPSS system, the issue of sensor node localization plays a critical role in improving computational efforts. To improve the performance of WSN in wireless CPSS further, this article presents a novel NN learning scheme, named RHOP-ELM-RCC, through the combination of DV-HOP and RSSI using ELM-RCC. It is effective while calculating the coordinates of unknown nodes and decreasing the location error with no additional hardware consumption. During the localization process, we combine DV-HOP with RSSI to reduce the distance error. When the distances between anchor nodes and unknown nodes have been obtained, the SLFN based on ELM is adopted to compute the coordinates of unknown nodes. Since ELM has good abilities of nonlinear mapping and fast learning, the localization accuracy of RHOP-ELM is significantly improved. However, as the original ELM is implemented on the basis of the measure, i.e., MSE, which is sensitive to outliers, the ELM-RCC is accordingly employed using RCC, which is robust against noises, and the half-quadratic technique is utilized for the optimization. Through the simulation comparisons for the localization error, the results show the satisfactory performance of our proposed scheme. Furthermore, the performance of RHOP-ELM-RCC is better than that of RHOP-ELM and other traditional localization schemes.

This article only focuses on the optimization of the localization algorithm. In the future, to further improve the localization performance while using our scheme in the real world, we will discuss some issues, e.g., modeling the sleep/active functional modes of sensors to manage the battery efficiently, optimizing our scheme with unknown transmission power and many others.

## Figures and Tables

**Table 1:** Simulation parameters.

Item	Value
area	50m×50m, 100m×100m
transmission range, *R*	25 m
path loss exponent, *τ*	4
transmitting power, $P_{\mathrm{tr}}$	0 dB
path loss of the reference, $P_{\mathrm{loss}}\left(d_{0}\right)$	−55 dB ($d_{0}$ = 1 m)
numbers of total nodes	50, 100, 120
ratios of anchors	10%, 20%, 30%, 40%, 50%
numbers of nodes in hidden layer	number of total nodes × ratio of anchors
numbers of RSSI samples	1, 5, 10, 15, 20
noise standard deviation	2, 5, 8, 11, 14
the proportion of outliers	0%, 3%, 6%, 9%, 12%, 15%, 18%
the threshold, *ε*	$10^{-3}$, $10^{-4}$
